# An American Text-Book of Surgery

**Published:** 1896-05

**Authors:** 


An American Text-Book of Surgery.—For Practitioners and Students. By Charles H. Burnett, M. D., Phineas S. Conner, M. D., Frederic S. Dennis, M. D., William W. Keen, M. D., Charles B. Nancrede, M. D., Roswell Park, M. D., Lewis S. Pilcher, M. D., Nicholas Senn, M. D., Francis J. Shepherd, M. D., Lewis A. Stimson, M. D., William Thomson, M. D., J. Collins Warren, M. D., and J. William White, M. D. Edited by William W. Keen, M. D., LL. D., and J. William White, M. D., Ph. D. Second Edition, carefully Revised. Royal Octavo; 1248 pages. Price in cloth, $7.00; Sheep, $8.00; Half-Russia, $9.00. For sale by subscription only. W. B. Saunders, Publisher, 925 Walnut Street, Philadelphia. 1895.

The cordial reception of the first edition of this book by the medical profession of this and other English speaking countries, together with its adoption as a text-book by more than sixty of the medical colleges of the United States, indicates pretty clearly the superior character of the book. Notwithstanding it has been only about three years since the first edition was issued from the press, this edition has been thoroughly revised, more pages have been added, and about thirty new illustrations have been inserted. Among the many new subjects treated of in this second edition are, a new section on acromegaly; the use of Murphy's button in intestinal anastomosis; the effects of modern small arms in military surgery; the Hartley-

Krause method of removing the Gasserian ganglion; Witzel's method for gastrostomy; Schede's operation for thoracoplasty; the osteoplastic method of cranial resection; castration for enlarged prostate; Macewen's method of compressing the aorta in amputation at the hip joint, and a chapter on symphyseotomy. Much matter has been added to the sections dealing with fractures, dislocations, appendicitis, uterine displacements, amputations of the breast, etc.

Many of the illustrations have been redrawn and about thirty new cuts have been added.

The book covers the whole field of surgery as taught and practiced by the best surgeons of this country at this time, and we believe that no single volume work is superior to this one. For ready reference, in every day practice, and as a text-book for the student, it has no superior. H.

